# Enhancing Aesthetics and Functionality of the Teeth Using Injectable Composite Resin Technique

**DOI:** 10.7759/cureus.59974

**Published:** 2024-05-09

**Authors:** Pratik Rathod, Aditya Patel, Nikhil Mankar, Manoj Chandak, Anuja Ikhar

**Affiliations:** 1 Department of Conservative Dentistry and Endodontics, Sharad Pawar Dental College and Hospital, Datta Meghe Institute of Higher Education and Research, Wardha, IND

**Keywords:** composite veneer, composite resin injection, transparent silicone index, functional rehabilitation, esthetic rehabilitation, injectable composite

## Abstract

In order to prepare composite restorations without the requirement for tooth preparation, the injectable composite resin technique uses a clear silicone index in a minimally invasive direct approach to imitate a diagnostic wax-up. This case report features a 34-year-old female patient having aesthetic and functional concerns, notably spacing between the teeth, insufficient tooth visibility, and diastema in the upper anterior region. Upon clinical examination, spacing and midline diastema were observed. The maxillary incisors and canines were to have composite veneers made as part of the treatment plan. A wax-up and transparent silicone index was prepared following the assessment of a try-in of the prepared mock-up. Subsequenty, for restoring the teeth, a clear silicone index was used to inject and polymerize the flowable composite. The desired outcomes included elongated teeth to enhance visibility, closure of diastemas, and reshaping of the canines. Over a 12-month follow-up period, the patient exhibited no signs of soft tissue inflammation or significant wear. The described technique is characterized by its minimal invasiveness, cost-effectiveness, and suitability for definitive and provisional restorations. Desirable results can be achieved by appropriate planning and adhering to a meticulous planning while minimizing tooth structure loss.

## Introduction

The goals of contemporary dental rehabilitation procedures are both functional and aesthetic. The process of making direct or indirect final restorations is still very tedious and time-consuming, demanding highly specific skills and attention to meticulous details from the clinician [1]. It takes a lot of clinical work to finish the conventional direct anterior restorative placement procedure. It frequently entails a laborious layering procedure, which restricts their optimal indication to a small number of restorations at once. Furthermore, because predefined tooth shapes or contours are not used in traditional direct restorations, the process is less predictable and depends on the operator. When multiple anterior restorations or a smile change is required, indirect restorations are preferred by clinicians [2]. However, a dental technician is usually assigned to fabricate indirect restorations, which increases the cost and delays delivery.

Silicone indices are incredibly helpful in modern restorative dentistry, which ranges from the treatment planning to the tooth preparation and final restorative stages. The injection technique using flowable composite suggests a quick and simple way to restore the contours and shape of worn-out/defective teeth [3-5]. The intended result is replicated on the model by creating a wax-up after an impression is taken and filled with stone. This wax-up is imprinted using a transparent polyvinyl siloxane (PVS) substance. The teeth that need to be restored have small access holes made facially to their incisal margins. Alternate teeth undergo etching and bonding, while the rest are isolated using polytetrafluoroethylene tape. For the prepared and photopolymerized teeth, the injection of flowable composite takes place via the access holes after the PVS mold has been inserted and adjusted to provide a perfect fit. The remaining isolated teeth go through this procedure once again, and once the mold has been removed, the final restorations are polished and finished. The distance between the PVS mold and the natural tooth is inevitable since it mimics the entire wax-up process. If flowable composite is injected into one tooth space, it may leak into the adjacent space and polymerize on that tooth. This can be challenging to clean, and it takes time to reshape the teeth, which defeats the very goal of this technique saving time while producing a desirable outcome. Comparing the injectable composite resin approach to traditional ceramic veneer operations, it is less intrusive and costs less money [6]. This report details the effective application of this approach, with some adjustments, in the case of a 34-year-old female patient who had reduced tooth visibility and diastema-related functional and aesthetic issues.

## Case presentation

**Figure 1:**
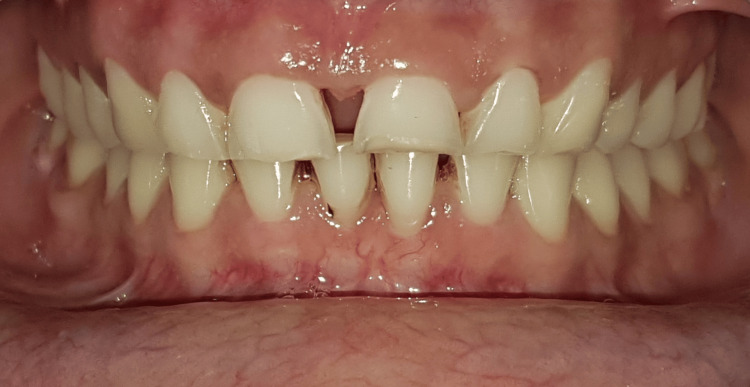
Preoperative image Preoperative image showing wearing of the teeth and spacing in the anterior teeth region.

A 34-year-old female patient reported to the Sharad Pawar Dental College and Hospital in Wardha with complaints of diastema and insufficient tooth visibility, leading to functional and aesthetic issues. The patient expressed dissatisfaction with her smile, and her anterior teeth showed spacing and wearing of teeth incisally (Figure 1).

Different treatment alternatives were reviewed, and aesthetic rehabilitation was advised. First, ceramic veneers were suggested to be made and applied, which offers superior aesthetics with precise color matching and durability. Alternatively, the second option involved composite veneers, a more cost-effective solution with potentially less optimal aesthetics and longevity. The patient opted for composite veneers due to their affordability. The injectable composite resin approach was chosen due to the requirement for functional rehabilitation. This method ensured the exact restoration of both function and aesthetics by enabling replication of diagnostic wax-up into restorations intraorally.

**Figure 2:**
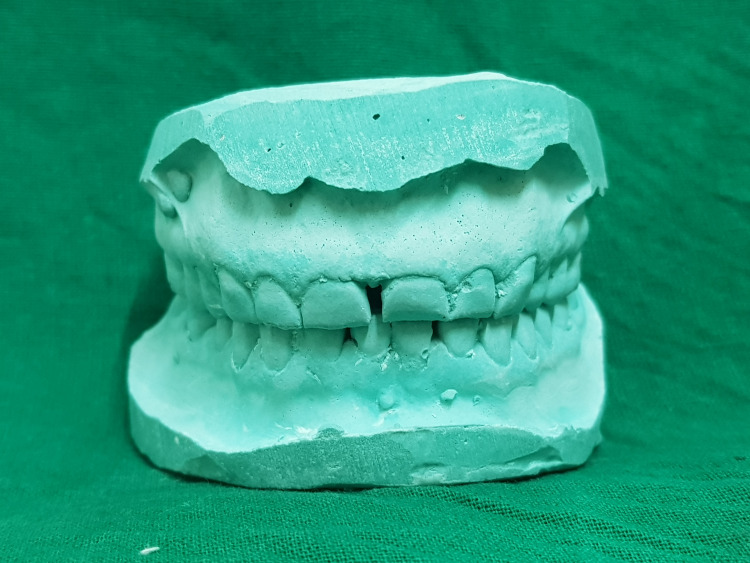
Preoperative Diagnostic Cast Diagnostic casts meticulously reflect the condition of worn teeth.

During the initial appointment, impressions and photographs were obtained for detailed analysis, and dental casts were subsequently prepared (Figure 2).

**Figure 3:**
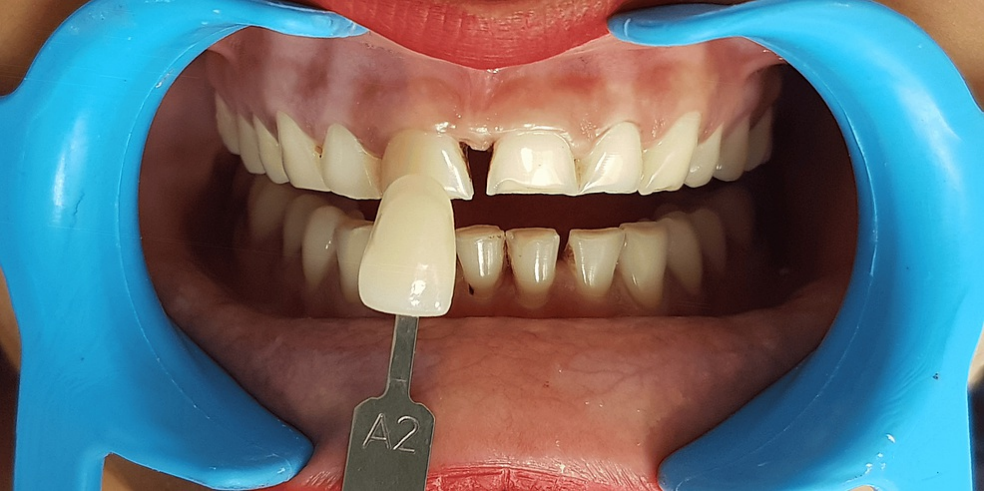
Shade selection Using Vita shade guide, the selection of shade was done.

Shade selection was done using the Vita shade guide (Vita classical A1-D4 shade guide, Vita Zahnfabrik). The choice of A2 shade was made for both the incisors and canines (Figure 3).

**Figure 4:**
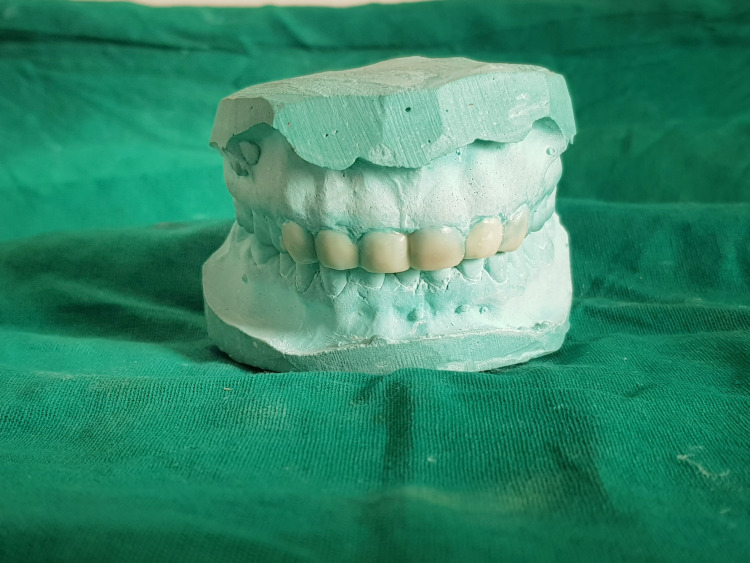
Prepared wax-up on cast Wax-up was done on the diagnostic cast for replication of prepared wax-up into restorations intraorally.

A wax-up using inlay wax (type 2) was meticulously crafted following meticulous analysis, adhering to aesthetic principles and relevant parameters (Figure 4).

The treatment plan involved the preparation of six composite veneers, featuring classic composite veneers having A2 shade for both the canines and incisors. To ensure optimal outcomes, restoration margins were positioned supragingivally (0.5 mm) to avoid the risk of gingival inflammation in the future. Notably, the decision to opt for supragingival margins was supported by the patient's low lip line and the fact that the gingival margins remained concealed during a full smile, thus preserving the overall aesthetic harmony.

The wax-up underwent a final verification in the oral cavity by preparing a mock-up of composite veneers using resin-based provisional material. This involved utilizing a silicone index composed of PVS combined with A2 shade resin-based provisional material. Both the patient and clinician approved all parameters during this stage. After that, a final transparent silicone index was made ready for injecting the flowable composite intraorally.

To initiate this process, an impression of the wax-up was taken using transparent PVS (Memosil 2, Heraeus Kulzer) loaded onto a nonperforate tray. Subsequently, the clear silicone was delicately positioned over the prepared wax-up before being affixed to the tray.

**Figure 5:**
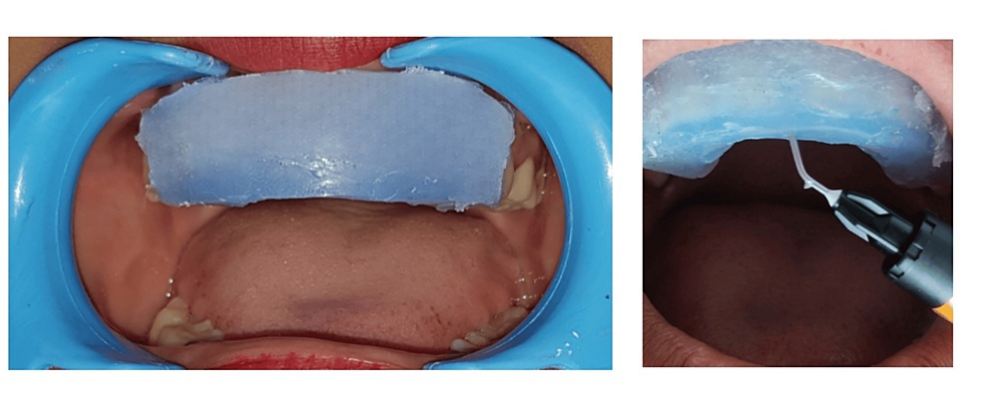
Intraoral placement of clear silicone index

Afterward, the cast was carefully positioned within the tray. Once the transparent silicone had fully polymerized, any surplus material was meticulously trimmed away by following the contours of the sulcus margins using a scalpel. Rotary burs were used to establish openings along the incisal edges and facilitate the injection of flowable composite material. After meticulously removing all surface stains and plaques, each tooth was carefully restored to ensure the formation of enough contact. Etching of the tooth followed by rinsing was done. Following this, a dental cord (Roeko Stay-Put, Coltene) was packed into the sulcus to prevent subgingival composite flow mechanically. Isolation of adjacent teeth was done using polytetrafluoroethylene tape. Application of adhesive (3M Adper Single Bond 2, ESPE) was done. The proper positioning and placement of clear silicone index was done intraorally (Figure 5 ).

Insertion of the flowable nanohybrid composite (Beautifil Injectable, Shofu, Japan) was done through the prepared holes on the incisal edge. The composite was carefully injected into the space between the tooth and silicone index to ensure complete coverage. It was then flash-polymerized using an LED curing light for 3 seconds. Any excess material in the sulcular area was meticulously removed using a probe. Subsequently, definitive light curing was done for 20 seconds through a transparent index from both the buccal and incisal surfaces. To prevent the development of an oxygen-inhibited layer, glycerin gel was applied to every surface. After removing the silicone index, additional light curing was applied for 20 seconds to facilitate the polymerization of the oxygen-inhibited layer, ensuring comprehensive and durable restoration.

The dental cord and polytetrafluoroethylene tape were carefully removed once polymerization was complete. The veneers were then polished using a handpiece, interproximal strips, polishing rubbers, and brushes loaded with polishing paste to produce a glossy appearance. This meticulous polishing process aimed to prevent plaque accumulation and staining, ensuring long-term aesthetic appeal. Furthermore, the margins of the restorations were precisely positioned as planned, maintaining their supragingival positions as intended. The teeth were successfully elongated and reshaped in accordance with the predetermined treatment plan, resulting in the desired aesthetic and functional improvements (Figure 7).

**Figure 6:**
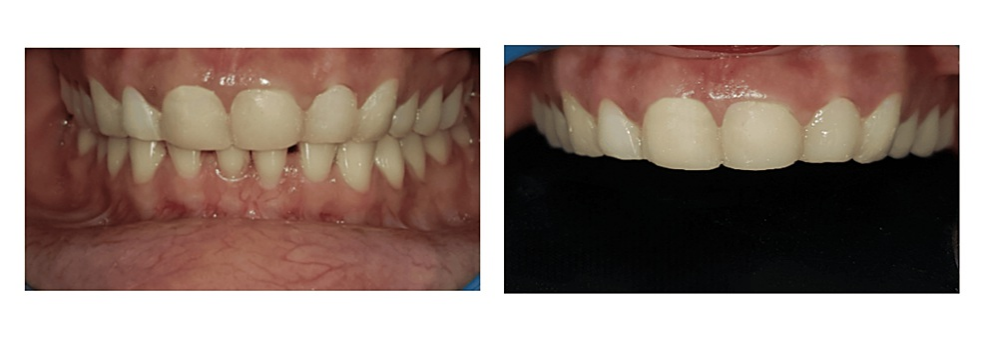
Postoperative image after treatment Image showing the restored teeth using injectable composite.

Follow-up was conducted for a duration of 12 months. The patient was scheduled for routine follow-up sessions every three months. During these visits, there was no discernible wear, postoperative sensitivity, blood upon probing, or soft tissue inflammation (Figure 8).

**Figure 7:**
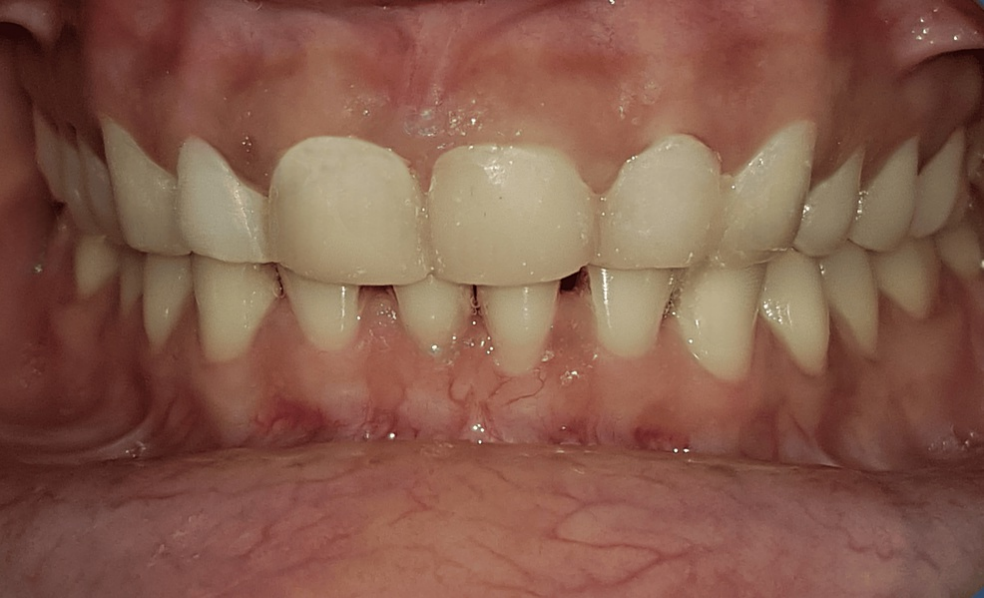
At 12-month follow-up

## Discussion

This case study describes the effective use of injectable composite resin in treating an adult patient's diastemas and restricted tooth visibility. This approach has several advantages over conventional ceramic veneer procedures, including less clinical time, reduced cost, and less disruption of the tooth's structural integrity. It can also be used as a therapeutic technique that is only additive. Furthermore, the process is reversible and involves a straightforward adhesive approach, making it suitable for restoring worn and chipped teeth [7].

The mechanical characteristics, strength, wear resistance, polishability, translucency, and other qualities of flowable composites have all significantly improved in recent years [8]. Flowable composites have advanced significantly over time, exhibiting superior placement qualities and marginal adaption, reducing voids because of their exceptional wettability on various substrates. Their reduced elastic modulus and stress-buffering capacity further enhance their superiority over traditional composites. Flowable composites effectively absorb significant compressive stresses brought on by tooth flexure, particularly in cervical restorations. These qualities extend their usefulness to composite veneers, which cover the whole tooth surface with a similar physical structure. This adaptability demonstrates how well flowable composites perform in various clinical restorative scenarios.

Recent meta-analyses have indicated no statistically or clinically significant differences between flowable and conventional composites in any assessed outcomes [9,10]. Flowable composites exhibit superb placement characteristics and marginal adaptation, resulting in fewer voids attributed to their superior wettability on various substrates. Furthermore, they display a lower elastic modulus and effective stress-buffering capability, surpassing traditional composites. High compression forces from tooth flexure impact cervical restorations; flowable composites can absorb these stresses. These properties can also be applied to composite veneers with a similar physical configuration covering the entire tooth surface [10-12]. Because flowable composite resin makes exact intraoral reproduction of the prepared wax-up easier, it is considered more appropriate for use with a transparent silicone index. In certain studies with traditional composite material, clinicians had to exert significant external pressure on the index to accurately replicate the tooth morphology [7]. Index and restoration distortion is less likely when a complete index without segments is well-stabilized and when the flowable composite is passively injected without external pressure. Flowable composite is the preferred material for injectable composite resin technology because of its excellent consistency for injection via the index, enhanced physical qualities compared to conventional composites, and good marginal adaption [11,12].

Utilizing PVS material for indexing represents an innovative approach, particularly beneficial in addressing demanding aesthetic cases and achieving satisfactory outcomes. The benefits of clear PVS material are its translucency for better visual control during the treatment, light-curing through the index and perfect adaptation to teeth. This clear silicone material offers absolute transparency, highly effective in managing complex clinical scenarios. The transparency of this clear silicone material enables precise visual control and promotes effective light-curing through the silicone. This leads to a higher conversion rate, reducing the formation of an oxygen inhibition layer and streamlining the final polishing process. Furthermore, combining the injection molding technique with the utilization of clear silicone material is optimal for addressing cases of wear or reconstructing intricate morphologies. The material's transparency enables accurate control during injection, even when treating multiple teeth simultaneously. The clear PVS index serves as a template for precisely adapting flowable composite into the space between the teeth and the index. Furthermore, injectable composites are well-suited for anterior instances due to their high flexural strength and wear resistance, ensuring long-term durability and successful aesthetic results.

In addition, if a PVS mold is used to duplicate the whole wax-up, a space between the mold and the original tooth is usually encountered. Flowable composite injection into this area carries the danger of leaking onto adjacent teeth, which could lead to polymerization and complicated cleaning. The process of reshaping the affected teeth can wind up taking a lot of time, which will ultimately undermine the technique's intended efficiency.

It's important to follow well-established principles such as the tooth width-to-length ratios and the golden ratio to get visually acceptable outcomes while considering tooth proportion recommendations. Patient perception is crucial since treatment decisions and satisfaction with the final result are influenced by the preferences and expectations of the patient. Aesthetic preferences are also influenced by cultural and socioeconomic considerations, which guide decisions about teeth size, shape, and symmetry. Furthermore, the dentist's artistic expertise and influences greatly influence the final restoration, guaranteeing that it satisfies the patient's aesthetic preferences and technical specifications [13].

## Conclusions

The direct composite veneer technique, which uses injectable composite materials, presents a straightforward but efficient treatment option for erosive-abrasive lesions. This approach presents several advantages, such as single-session treatment, pleasing aesthetic results, straightforward procedural steps, cost-effectiveness, and enduring outcomes over a 12-month follow-up period, particularly when accompanied by a comprehensive prevention and education program for the patient. Composite veneers created using the injectable composite resin technique are a valuable, efficient, and more cost-effective alternative to ceramic veneers, especially in cases similar to the one presented here. With meticulous planning and creating a detailed wax-up, functional and aesthetic objectives can be attained. This wax-up serves as a precise guide, facilitating the accurate transfer of the treatment plan into restorations with a clear index.
